# Supplementary material for: Realizing tight-binding Hamiltonians using site-controlled coupled cavity arrays
Source: Nat Commun. 2023 Aug 29;14:5260. doi: 10.1038/s41467-023-41034-x (PMC10465588; doi:10.1038/s41467-023-41034-x)
Supplement: Supplementary file 1 — Supplementary Information [file 41467_2023_41034_MOESM1_ESM.pdf]

Supplementary Information for

Realizing tight-binding Hamiltonians using site-controlled coupled cavity arrays

Authors: Abhi Saxena*, Arnab Manna, Rahul Trivedi, and Arka Majumdar*
Correspondence to: abhi15@uw.edu, arka@uw.edu

This file includes:

Methods
Section S1. Optical design & considerations
Section S2. Hamiltonian tomography
Section S3. Thermal crosstalk simulations
Section S4. Extracting the thermal crosstalk
Fig. S1. Fabrication flow.
Fig. S2. Waveguide mode profiles.
Fig. S3. Coupled waveguide mode profiles.
Fig. S4. Schematic depicting a general CCA device.
Fig. S5. Ellipsometry data of evaporated alumina.
Fig. S6. Thermal simulation results.
Fig. S7. Thermal crosstalk extraction.

Methods

Fabrication:

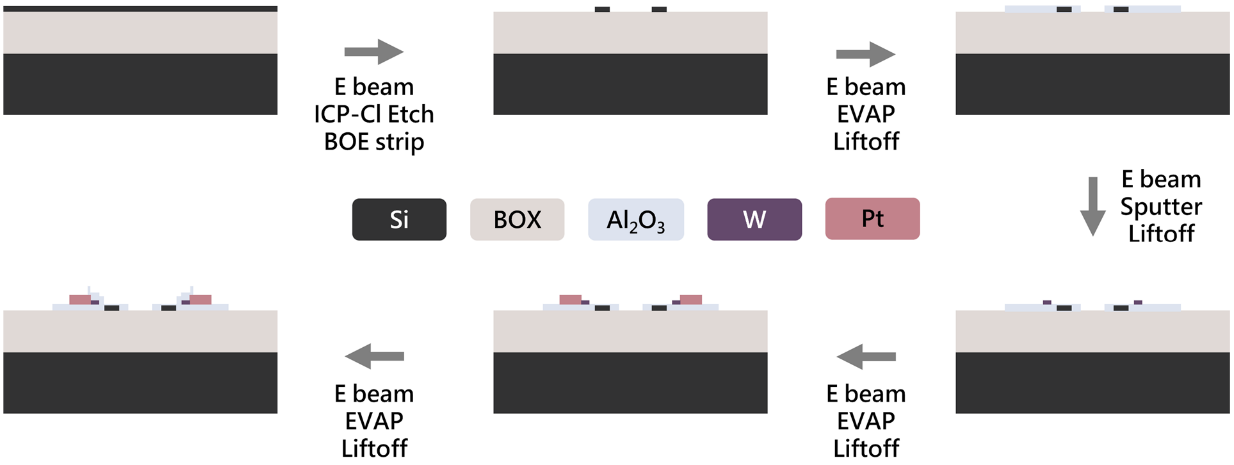

Fig. S1. Fabrication flow. The CCA was defined on a silicon on insulator (SOI) chip via electron-beam lithography using HSQ resist. Cl_2 gas was then used to etch the structures. The resist was then removed using dilute BOE. For the deposition steps positive tone $PMMA$ resist was used for patterning. Bottom Al_2O_3 layer is 265 nm thick and the sputtered tungsten (W) layer making up the heating elements is 150 nm thick. The contact pads are made up of 25 nm Ti / 325 nm Pt layers. The final Al_2O_3 cladding over the islands is 300 nm thick.

Supplementary Text:

Section S1: Optical design & considerations:

S1A: Waveguide Modes:

Our device consists of racetrack resonators fabricated on $220\,nm$ thick silicon on insulator platform. The resonators have two differing waveguide sections: (i) a standard ridge waveguide section which is $554\,nm$ wide with an 82° slant and (ii) and an embedded waveguide section where the resonator makes thermal contact with the heater through the bottom alumina layer of the islands. The simulated optical mode profile (using ANSYS Lumerical MODE) for both these sections is plotted in Fig. S2. The simulated waveguide losses are $9.2 \times 10^{-13} dB/cm$ and $7.8 \times 10^{-9}\ dB/cm$ respectively. As evident the loss is negligible even for the heater integrated design as we were able to utilize the bottom alumina platform (which is $265\,nm$ thick) to place the tungsten heater both above the plane of the optical mode and at a far enough ($1.5\ \mu m$) lateral distance to ensure minimal increase in the absorption losses.

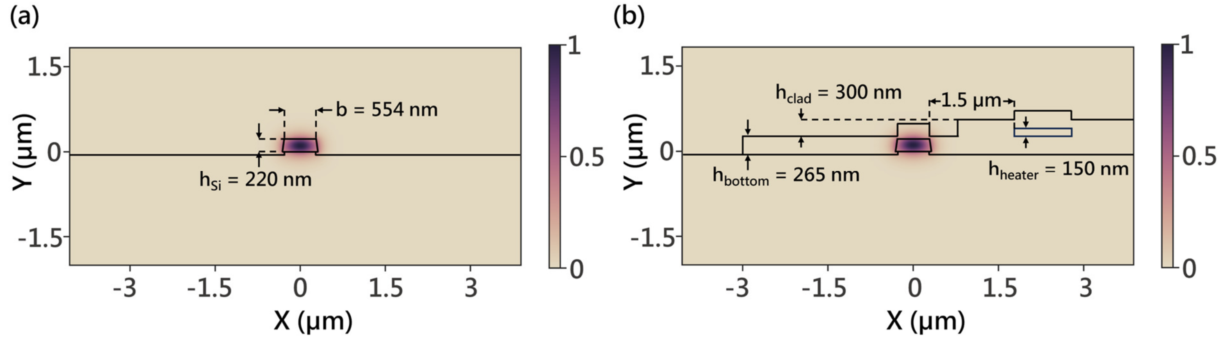

Fig. S2. Waveguide mode profiles ($|E|^2/|E|^2_{max}$). (a) Profile of the waveguide mode in the sections without any cladding layers. The effective index of the mode is $n_{eff} = 2.556$ and the group index is $n_g = 4.13$. The simulated loss is $9.213 \times 10^{-13}\ dB/cm$. A lateral undercut of $\sim 27\,nm$ occurs during the BOE strip of HSQ resist. (b) Profile of the waveguide mode where resonator makes thermal contact with the island heaters with the tungsten heating element being $1\ \mu m$ wide. The effective index of the mode is $n_{eff} = 2.61$ and the group index is $n_g = 4.006$. The simulated loss is $7.75 \times 10^{-9}\ dB/cm$.

S1B: Hopping rates:

Another aspect of the optical design deals with the interplay of the physical spacing between the resonators and the corresponding hopping rates between the sites. As the waveguides forming the coupling region of neighboring resonators in a CCA lie in close proximity, their respective modes overlap with each other to create two coupled modes as depicted in Fig. S3. Their coupling constant k can be estimated numerically by simulating the eigenmodes of the two adjacent waveguides and is given by the following relation:

$$k = sin\left(\frac{\pi\Delta n_{eff}}{\mu^{\lambda}} L_{coupling}\right) \tag{S1}$$

where Δn_{eff} is the difference between the effective indices of the coupled waveguide modes, $L_{coupling}$ is the coupling length between the resonators and μ^{λ} is the resonant wavelength of the resonators. Given the coupling constant between the coupled waveguide sections, the corresponding hopping rate between the resonators (assuming no disorder) can then be calculated using the following relation[1,2] :

$$J = \frac{k v_g}{L_{resonator}} \quad \text{(S2)}$$

where v_g denotes the group velocity of the waveguide mode and the $L_{resonator}$ denotes the length of resonator. Combing these two equations, the estimated hopping rate between the resonators can be written as

$$J = sin\left(\frac{\pi \Delta n_{eff}}{\mu^{\lambda}} L_{coupling}\right) \frac{c}{n_g L_{resonator}} \quad \text{(S3)}$$

Note that we have substituted $v_g = c/n_g$ where n_g denotes the group index of the waveguide mode and the hopping rate J is obtained in units of rad/s.

Substituting for the values we get $J \approx 69.1\ GHz$. From our tomography algorithm the experimentally measured hopping rates between the resonators in the CCA were found to be $72.1\ GHz$, $75.7\ GHz$, $75.1\ GHz$, $77.9\ GHz$, $69.5\ GHz$, $70\ GHz$, $64.5\ GHz$ giving a mean hopping rate of $J_{mean} \approx\ 72\ GHz$ with a standard deviation of $\sigma_J = 4.2\ GHz$. Additionally, on the same chip we fabricated two photonic molecules ($N = 2$) and the reconstructed hopping rate for these after measurement and running the tomography algorithm, was found to be $68.2\ GHz$ and $68.8\ GHz$ respectively. We note that even though the estimated hopping rate is in close agreement of the measured values, relation S3 is strictly true only in absence of onsite disorder. Additionally, the hopping rates are very sensitive to minor changes in difference of refractive indices of the coupled modes (for small k, $J \propto\ \Delta n_{eff}$) which can arise due to a variety of fabrication induced local variations. As nanofabrication processes are always susceptible to inherent disorder, the above strongly emphasizes the need of using the tomography algorithm to accurately reconstruct the actual hopping rates (and onsite potentials) of the device post-fabrication.

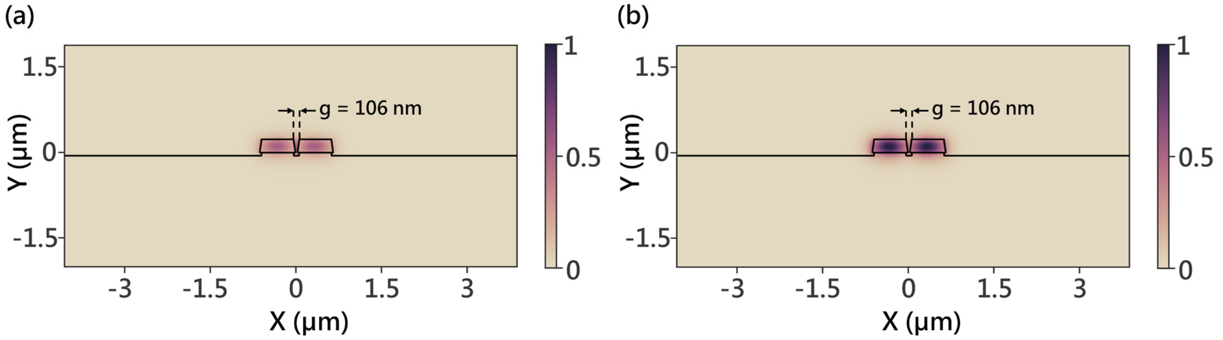

Fig. S3. Coupled waveguide mode profiles ($|E|^2/|E|^2_{max}$). (a) Mode profile of the first coupled waveguide mode $n_{eff} = 2.568$. (b) Mode profile of the second coupled waveguide mode, $n_{eff} = 2.5525$. $\Delta n_{eff} \approx 0.0155$.

Section S2: Hamiltonian Tomography:

For a coupled cavity array with N racetrack resonators with grating couplers at first and last sites, the non-interacting system Hamiltonian H can be written as

$$H = \sum_n \mu_n a_n^\dagger a_n + J_n \left(a_{n+1}^\dagger a_n + a_n^\dagger a_{n+1}\right) \tag{S4}$$

where a_n denotes onsite destruction operator, μ_n denotes the onsite potential and J_n denotes the hopping rates between n^{th} and $(n+1)^{th}$ sites. Further using input-output formalism we can write:

$$\begin{aligned} \dot{a_0} &= -j(\mu_0 a_0 + J_0 a_1) - \frac{\kappa_0}{2} a_0 - \frac{\gamma_0}{2} a_0 - \sqrt{\gamma_0}\, x_0, \\ \dot{a_n} &= -j(\mu_n a_n + J_{n-1} a_{n-1} + J_n a_{n+1}) - \frac{\kappa_n}{2} a_n, where\ n = 1,2 \ldots N-2 \\ \dot{a_{N-1}} &= -j(\mu_{N-1} a_{N-1} + J_{N-2} a_{N-2}) - \frac{\kappa_{N-1}}{2} a_{N-1} - \frac{\gamma_{N-1}}{2} a_{N-1} \end{aligned} \tag{S5}$$

where κ_n denotes the loss rate to environment at each site, γ_n denotes the coupling rate to the grating couplers, x_0 denotes the destruction operator of the input into the system. The system can be visualized with the aid of the schematic depicted in Fig. S4.

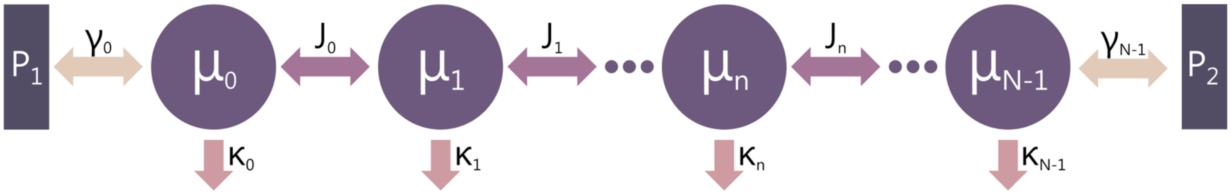

Fig. S4. Schematic depicting a general CCA device. Each resonator site is characterized by the onsite potential μ_n and the loss rate to the environment/absorption is denoted by κ_n. The hopping rates between the sites are denoted by J_n. The array is probed using grating couplers coupled to the first and last sites of the array with coupling rates denoted by γ_n. The grating couplers are themselves denoted by the ports P_1, P_2.

Taking Fourier transform on these equations gives:

$$\begin{aligned} -j\omega a_0 &= -j(\mu_0 a_0 + J_0 a_1) - \frac{\kappa_0}{2} a_0 - \frac{\gamma_0}{2} a_0 - \sqrt{\gamma_0}\, x_0, \\ -j\omega a_n &= -j(\mu_n a_n + J_{n-1} a_{n-1} + J_n a_{n+1}) - \frac{\kappa_n}{2} a_n, where\ n = 1,2 \ldots N-2 \\ -j\omega a_{N-1} &= -j(\mu_{N-1} a_{N-1} + J_{N-2} a_{N-2}) - \frac{\kappa_{N-1}}{2} a_{N-1} - \frac{\gamma_{N-1}}{2} a_{N-1} \end{aligned} \tag{S6}$$

Rearranging the terms gives:

$$\sqrt{\gamma_0}\,x_0 = j\left(\omega - \left(\mu_0 - j\frac{\kappa_0}{2} - j\frac{\gamma_0}{2}\right)\right)a_0 - j(J_0 a_1),$$

$$0 = j\left(\omega - \left(\mu_n - j\frac{\kappa_n}{2}\right)\right)a_n - j(J_{n-1}a_{n-1} + J_n a_{n+1}), where\ n = 1,2 \dots N-2 \qquad \text{(S7)}$$

$$0 = j\left(\omega - \left(\mu_{N-1} - j\frac{\kappa_{N-1}}{2} - j\frac{\gamma_{N-1}}{2}\right)\right)a_{N-1} - j(J_{N-2}a_{N-1})$$

Writing as a matrix:

$$j\begin{pmatrix} \omega - \left(\mu_0 - j\frac{\kappa_0}{2} - j\frac{\gamma_0}{2}\right) & -J_0 & 0 & 0 & \dots \\ -J_0 & \omega - \left(\mu_n - j\frac{\kappa_n}{2}\right) & -J_1 & 0 & \dots \\ \vdots & \ddots & \ddots & \ddots & \vdots \\ \dots & \dots & 0 & -J_{N-2} & \omega - \left(\mu_{N-1} - j\frac{\kappa_{N-1}}{2} - j\frac{\gamma_{N-1}}{2}\right) \end{pmatrix}\begin{pmatrix} a_0 \\ a_1 \\ \vdots \\ a_{N-1} \end{pmatrix} = \begin{pmatrix} \sqrt{\gamma_0}x_0 \\ 0 \\ \vdots \\ 0 \end{pmatrix} \qquad \text{(S8)}$$

Further we can define H_{eff} as

$$H_{eff} = \begin{pmatrix} \mu_0 - j\frac{\kappa_0}{2} - j\frac{\gamma_0}{2} & J_0 & 0 & 0 & \dots \\ J_0 & \mu_1 - j\frac{\kappa_1}{2} & J_1 & 0 & \dots \\ \vdots & \ddots & \ddots & \ddots & \vdots \\ \dots & \dots & 0 & J_{N-2} & \mu_{N-1} - j\frac{\kappa_{N-1}}{2} - j\frac{\gamma_{N-1}}{2} \end{pmatrix}, |x_{in}\rangle = \begin{pmatrix} \sqrt{\gamma_0}x_0 \\ 0 \\ \vdots \\ 0 \end{pmatrix} \qquad \text{(S9)}$$

This allows us to compress Eq. S8 into

$$j\left(\omega\mathbb{I} - H_{eff}\right)|a_n\rangle = |x_{in}\rangle \qquad \text{(S10)}$$

Hence, we obtain $|a_n\rangle$ as

$$|a_n\rangle = -j\left(\omega\mathbb{I} - H_{eff}\right)^{-1}|x_{in}\rangle \qquad \text{(S11)}$$

From input-output formalism we can also write the output modes as:

$$|y_{out}\rangle = |x_{in}\rangle + \left|\sqrt{\gamma_n}a_n\right\rangle \qquad \text{(S12)}$$

Separately, we know that the two experimentally measurable quantities from the device the reflection spectrum $|R(\omega)|^2$ and the transmission spectrum $|T(\omega)|^2$ of the system can be written as:

$$|R(\omega)|^2 = \left|\frac{y_0}{x_0}\right|^2, |T(\omega)|^2 = \left|\frac{y_{N-1}}{x_0}\right|^2 \qquad \text{(S13)}$$

Substituting for y_0 from Eq. S11, S12 we can obtain $|R(\omega)|^2$ as

$$|R(\omega)|^2 = \left|\frac{y_0}{x_0}\right|^2 = \left|\frac{x_0 - j\gamma_0\, x_0 \langle v_0|\left(\omega\mathbb{I} - H_{eff}\right)^{-1}|v_0\rangle}{x_0}\right|^2 = \left|1 - j\gamma_0 \left\langle v_0 \left|\frac{1}{\omega\mathbb{I} - H_{eff}}\right| v_0\right\rangle\right|^2 \quad \text{(S14)}$$

where $|v_0\rangle = \begin{pmatrix} 1 \\ 0 \\ \vdots \\ 0 \end{pmatrix}$.

Let $\epsilon_\alpha, |\epsilon_\alpha\rangle$ denote the eigenvalues and eigenvectors of H_{eff}, as H_{eff} is complex symmetric matrix we can write

$$H_{eff} = \sum_\alpha \epsilon_\alpha |\epsilon_\alpha\rangle\langle\epsilon_\alpha| \quad \text{(S15)}$$

Note that $\langle\epsilon_\alpha|$ denotes the transpose and not the conjugate transpose of $|\epsilon_\alpha\rangle$ and $\langle\epsilon_\beta|\epsilon_\alpha\rangle = \delta_{\beta\alpha}$. This also implies that we can expand $\left(\omega\mathbb{I} - H_{eff}\right)^{-1}$ as:

$$\frac{1}{\omega\mathbb{I} - H_{eff}} = \sum_\alpha \frac{|\epsilon_\alpha\rangle\langle\epsilon_\alpha|}{\omega - \epsilon_\alpha} \quad \text{(S16)}$$

then we can write Eq. S14 as

$$|R(\omega)|^2 = \left|1 - j\gamma_0 \sum_\alpha \langle v_0| \frac{|\epsilon_\alpha\rangle\langle\epsilon_\alpha|}{\omega - \epsilon_\alpha} |v_0\rangle\right|^2 = \left|1 - j\gamma_0 \sum_\alpha \frac{\langle v_0|\epsilon_\alpha\rangle^2}{\omega - \epsilon_\alpha}\right|^2 \quad \text{(S17)}$$

Similarly, for transmission spectrum $|T(\omega)|^2$ we have

$$|T(\omega)|^2 = \left|\frac{y_{N-1}}{x_0}\right|^2 = \left|\frac{-j\sqrt{\gamma_0\gamma_{N-1}}\, x_0\langle v_{N-1}|\left(\omega\mathbb{I} - H_{eff}\right)^{-1}|v_0\rangle}{x_0}\right|^2$$
$$= \left|-j\sqrt{\gamma_0\gamma_{N-1}} \left\langle v_{N-1}\left|\frac{1}{\omega\mathbb{I} - H_{eff}}\right| v_0\right\rangle\right|^2 \quad \text{(S18)}$$

Using Eq. S16 we can write:

$$|T(\omega)|^2 = \left|-j\sqrt{\gamma_0\gamma_{N-1}} \sum_\alpha \langle v_{N-1}| \frac{|\epsilon_\alpha\rangle\langle\epsilon_\alpha|}{\omega - \epsilon_\alpha} |v_0\rangle\right|^2 = \left|-j\sqrt{\gamma_0\gamma_{N-1}} \sum_\alpha \frac{\langle v_{N-1}|\epsilon_\alpha\rangle\langle v_0|\epsilon_\alpha\rangle}{\omega - \epsilon_\alpha}\right|^2 \quad \text{(S19)}$$

Going further, exploiting the geometry of the device allows us to write

$$H_{eff}|v_n\rangle = \tilde{\mu}_n|v_n\rangle + J_{n-1}|v_{n-1}\rangle + J_n|v_{n+1}\rangle, \text{ where } \tilde{\mu}_n = \mu_n - j\frac{\kappa_n}{2} - j\frac{\gamma_n}{2}; \quad \text{(S20)}$$

$$|v_n\rangle = \begin{pmatrix} 0 \\ \vdots \\ 1 \\ \vdots \\ 0 \end{pmatrix} n^{th} row$$

Taking transpose and multiplying from right by $|\epsilon_\alpha\rangle$ we get

$$\langle v_n|H_{eff}|\epsilon_\alpha\rangle = \langle v_n|\tilde{\mu}_n|\epsilon_\alpha\rangle + \langle v_{n-1}|J_{n-1}|\epsilon_\alpha\rangle + \langle v_{n+1}|J_n|\epsilon_\alpha\rangle \tag{S21}$$

$$\Rightarrow \langle v_n|\epsilon_\alpha|\epsilon_\alpha\rangle = \langle v_n|\tilde{\mu}_n|\epsilon_\alpha\rangle + \langle v_{n-1}|J_{n-1}|\epsilon_\alpha\rangle + \langle v_{n+1}|J_n|\epsilon_\alpha\rangle \tag{S22}$$

$$\Rightarrow (\epsilon_\alpha - \tilde{\mu}_n)\langle v_n|\epsilon_\alpha\rangle - J_{n-1}\,\langle v_{n-1}|\epsilon_\alpha\rangle = J_n\,\langle v_{n+1}|\epsilon_\alpha\rangle \tag{S23}$$

Also using Eq. S15 we have,

$$\tilde{\mu}_n = \langle v_n|H_{eff}|v_n\rangle = \sum_\alpha \epsilon_\alpha \langle v_n|\epsilon_\alpha\rangle^2 \tag{S24}$$

Now we have all the ingredient equations needed for determining H_{eff}. The general method we use for Hamiltonian tomography can be summarized as an algorithm SA1:

Tomography algorithm

(i) Measure reflection spectrum $|R(\omega)|^2$ of the device and fit it to a sum N complex Lorentzian functions to obtain all the ϵ_α's and $\langle v_0|\epsilon_\alpha\rangle$'s using Eq. S17.
(ii) For the first site ($n = 0$), obtain the diagonal element $\tilde{\mu}_n$ of H_{eff} using Eq. S24. (SA1)
(iii) Normalize Eq. S23 to then obtain off diagonal element J_n.
(iv) Use the value of J_n to obtain all the $\langle v_{n+1}|\epsilon_\alpha\rangle$s needed for the next iteration.
(v) Repeat steps 3, 4, 5 for $n > 0$ until the entire H_{eff} has been determined.

We next demonstrate how the algorithm SA1 looks in practice:
(i) We begin with fitting the experimentally measured reflection spectrum $|R(\omega)|^2$ as sum of N Lorentzians to obtain the values of $\gamma_0, \langle v_0|\epsilon_\alpha\rangle, \epsilon_\alpha$ as

$$|R(\omega)|^2 = \left|1 - j\sum_\alpha \frac{A_\alpha e^{j\phi_\alpha}}{\omega - (\omega_\alpha - j\beta_\alpha)}\right|^2, where\ \epsilon_\alpha = \omega_\alpha - j\beta_\alpha, \quad \gamma_0\langle v_0|\epsilon_\alpha\rangle^2 = A_\alpha e^{j\phi_\alpha} \tag{S25}$$

From normalization it also follows that $\sum_\alpha \langle v_0|\epsilon_\alpha\rangle^2 = 1 \Rightarrow \sum_\alpha A_\alpha e^{j\phi_\alpha} = \gamma_0$. Here, fitting for the complex Lorentzians from the reflection spectrum $|R(\omega)|^2$ is done by minimizing the following expression to ensure that the finally fitted H_{eff} falls in the realm of physical possibility:

$$Min\left\{\| \, |R(\omega)|^2{}_{predicted} - \, |R(\omega)|^2{}_{measured} \, \|^2 + \left|Im\left(\sum_{\alpha} A_\alpha e^{j\phi_\alpha}\right)\right| + \sum_i |Im(J_i)|\right\} \tag{S26}$$

(ii) For the first site ($n = 0$) we use Eq. S24 to obtain the diagonal element $\tilde{\mu}_0$ as

$$\tilde{\mu}_0 = \sum_{\alpha} \epsilon_\alpha \langle v_0|\epsilon_\alpha\rangle^2 , = \sum_{\alpha} (\omega_\alpha - j\beta_\alpha) A_\alpha e^{j\phi_\alpha}/\gamma_0 \tag{S27}$$

(iii) Next we determine the first off-diagonal element J_0 using Eq. S23 ($(\epsilon_\alpha - \tilde{\mu}_0)\langle v_0|\epsilon_\alpha\rangle = J_0\langle v_1|\epsilon_\alpha\rangle$) for $n = 0$ and normalizing for $\sum_\alpha \langle v_1|\epsilon_\alpha\rangle^2 = 1$, giving:

$$J_0 = \sqrt{\sum_{\alpha} ((\epsilon_\alpha - \tilde{\mu}_0)\langle v_0|\epsilon_\alpha\rangle)^2} = \sqrt{\sum_{\alpha} (\omega_\alpha - j\beta_\alpha - \tilde{\mu}_0)^2 A_\alpha e^{j\phi_\alpha}/\gamma_0} \tag{S28}$$

(iv) Finally, we can substitute the obtained J_0 back into Eq. S23 to obtain $\langle v_1|\epsilon_\alpha\rangle$s needed for the next iteration as:

$$\langle v_1|\epsilon_\alpha\rangle = \frac{(\epsilon_\alpha - \tilde{\mu}_0)\langle v_0|\epsilon_\alpha\rangle}{J_0} = \frac{(\omega_\alpha - j\beta_\alpha - \tilde{\mu}_0)\sqrt{A_\alpha e^{j\phi_\alpha}/\gamma_0}}{J_0} \tag{S29}$$

(v) Next for $n = 1$, carrying out steps (ii)-(iv) gives:

$$\tilde{\mu}_1 = \sum_{\alpha} \epsilon_\alpha \langle v_1|\epsilon_\alpha\rangle^2 , \qquad (\epsilon_\alpha - \tilde{\mu}_1)\langle v_1|\epsilon_\alpha\rangle - J_0\langle v_0|\epsilon_\alpha\rangle = J_1\langle v_2|\epsilon_\alpha\rangle \tag{S30}$$

normalizing for $\sum_\alpha \langle v_2|\epsilon_\alpha\rangle^2 = 1$ gives

$$J_1 = \sqrt{\sum_{\alpha} ((\epsilon_\alpha - \tilde{\mu}_1)\langle v_1|\epsilon_\alpha\rangle - J_0\langle v_0|\epsilon_\alpha\rangle)^2} , \tag{S31}$$

$$and \ \langle v_2|\epsilon_\alpha\rangle = \frac{(\epsilon_\alpha - \tilde{\mu}_1)\langle v_1|\epsilon_\alpha\rangle - J_0\langle v_0|\epsilon_\alpha\rangle}{J_1}$$

And so on for $n > 1$ we can keep iterating over the steps (ii)-(iv) until we obtain all the $\tilde{\mu}_n$, J_n where $n \in [0, \dots N-1]$. At the end of the algorithm we will have a successfully mapped effective Hamiltonian H_{eff} describing the device.

Couple of things to note in the algorithm are:

1. We assume that all J_n have the same sign, and that it is a valid assumption for our devices.
2. The algorithm SA1 works for determining H_{eff} when we fit the measured reflection spectrum $|R(\omega)|^2$ to a sum of complex Lorentzian functions to obtain the eigenvalues ϵ_α and spectral weights $\langle v_0|\epsilon_\alpha\rangle$. While we can also fit the transmission spectrum $|T(\omega)|^2$ to a sum of complex Lorentzian functions to obtain the eigenvalues ϵ_αs, as evident form Eq. S19 we cannot uniquely determine the involved spectral weights $\langle v_0|\epsilon_\alpha\rangle, \langle v_{N-1}|\epsilon_\alpha\rangle$ from such a fit.

S2A: Quality factor extraction & considerations:
The relevant Q-factors for us here are the Q-factors of the eigenmodes of the system which effect Hamiltonian dynamics and the number of modes we can address for a given hopping rate, and the intrinsic Qs of the cavities which act as a barometer of loss in our system due to integration with island heaters. These can be calculated as follows: In the step (i) of the tomography algorithm we fit each of the supermodes to a complex Lorentzian and hence obtain both their eigenfrequencies and linewidths. The following table shows the quality factor of each of the eigenmodes calculated using the relation:

$$Q_\alpha = \frac{\omega_\alpha}{2\beta_\alpha} \tag{S32}$$

where Q_α denotes the Q-factor of the eigenmode α.

Table. S1. Q-factors of eigenmodes. Quality factors of the 8 supermodes of the CCA. The average linewidth of the eigenmodes is $7.41GHz$ and the average Q-factor is $Avg(Q_\alpha) \sim 2.8 \times 10^4$.

Q_0	Q_1	Q_2	Q_3	Q_4	Q_5	Q_6	Q_7
32432	32380	28314	21609	20394	23738	31814	31746

Additionally, after going through the entire tomography algorithm, we know all the complex onsite potentials lying along the diagonal of the reconstructed Hamiltonian H_{eff}. Hence, estimating the intrinsic the Q-factors of the cavities not directly coupled to the grating couplers ($n \neq 0, N-1$) is straightforward as their complex potentials can be expressed as ($\tilde{\mu}_n = \mu_n - j\frac{\kappa_n}{2}, n \neq 0, N-1$). The intrinsic Q-factor can then be calculated as:

$$Q_{intrinsic} = \frac{Re(\tilde{\mu}_n)}{-2Im(\tilde{\mu}_n)} \; \forall \, n \neq 0, N-1 \tag{S33}$$

The following table then shows the intrinsic Qs for the realized CCA:

Table. S2. Q-factors of individual cavities. Intrinsic quality factors of the 6 internal cavities ($n \in [1,6]$).

Q_1	Q_2	Q_3	Q_4	Q_5	Q_6
49821	28467	32146	32148	31137	71166

Finally, as we also obtained the coupling rate to the grating coupler from the algorithm ($\gamma \approx 11.6\ GHz$), we can use it to estimate the average loss rate in the CCA from the following relation:

$$Avg(\kappa_n) = 2\left(-\sum_{n=0}^{N-1} Im(\tilde{\mu}_n) - \gamma\right) \tag{S34}$$

Then the typical intrinsic Q can be estimated as

$$Expected(Q_{intrinsic}) = \frac{Avg(Re(\tilde{\mu}_n))}{Avg(\kappa_n)} \quad \text{(S35)}$$

which for our device gives $Avg(\kappa_n) = 4.49\ GHz$, $Expected(Q_{intrinsic}) \approx 4.43 \times 10^4$.
We also fabricated some add-drop filters (single racetrack resonators coupled to grating couplers on either side) on the same chip and measured their Q-factors before integration of the island heaters. The measured data is presented in the table below:

Table. S3. Q-factors of single racetrack resonators (in add-drop configuration) before the addition of island heaters. Q_L denotes the loaded Q-factor, $Q_{intrinsic}$ denotes the unloaded Q-factor, BW denotes the total bandwidth = loss rate + 2 × coupling rate.

No.	Q_L	$Q_{intrinsic}$	$BW\ (GHz)$
1	8324	99920	23.96
2	8701	257775	22.90
3	8949	192177	22.28

The Q-factor of the add-drop filters post integration of the island heaters was found to be similar to the Q-factor reported above for the main device. For example, for the add-drop filter $No.3$ the Q-factors post integration were: $Q_L = 7073$, $Q_{intrinsic} = 45067$ and $BW = 28.18\ GHz$. The intrinsic Q-factor matches very well with the $Expected(Q_{intrinsic})$ calculated from the CCA device.

Considerations:

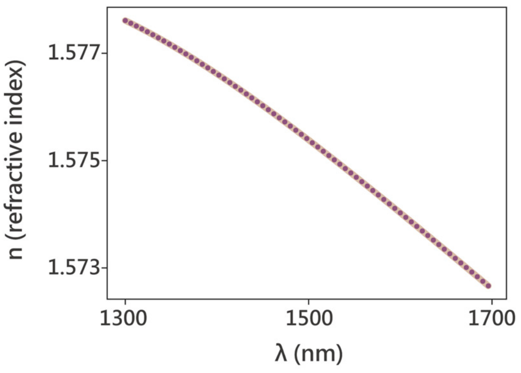

Fig. S5. Ellipsometry data of evaporated alumina. The refractive index of evaporated alumina is measured using ellipsometry. The imaginary part of the index is negligible, denoting that alumina is lossless in the window of operation of our CCA. $n(1505\ nm) = 1.57532$.

From our simulations in the above sections and measured data, we expect the major source of loss in our device to be the scattering loss due to mode mismatch between the cladded and uncladded sections of the racetrack resonator. As in subsection S1A, we used mode simulations to calculate the worst-case power coupling between the modes (by estimating their overlap) to be 0.99675; which is on the lower side, hence confirming our hypothesis. Additionally, based on our analysis we can perform a back of the hand calculation to estimate the kind of Q-factors we would

ultimately desire in order to realize very large-scale systems made up of say 1000 cavities. We know that the energy band of a $1D$ infinite tight-binding model is confined within $+2J$ to $-2J$ about the central potential[3]. As individual addressability to eigenmodes is desired, the maximum number of cavities that can be present given the average linewidth of the resonator can then be ball parked as $N_{cav} = \frac{4J}{Avg(\kappa_n)}$, where we assume small coupling rates to the input/output ports. We can then invert this equation to get an idea about the Q-factors desired for a CCA consisting of 1000 cavities:

$$Q_{desired} \sim N_{cav}\frac{\mu}{4J} = 1000\frac{199.29}{4 \times 0.072} \approx 6.9 \times 10^5$$

While this is an order of magnitude higher than the current reported Q-factor, in future works we aim to reduce the loss in the constituent cavities by designing better mode matched waveguide sections. We also plan to incorporate Euler bends[4] in our racetrack resonators and further optimize the fabrication processes to improve the Q-factors of our devices.

Section S3: Thermal crosstalk simulations:

For a racetrack resonator we know that $n_{eff}l = m\mu_n^\lambda$, where n_{eff} is the refractive index of the racetrack resonator, l is the length of the resonator, μ_n^λ is the onsite potential (in wavelength units) and $m \in Z$. Let a segment of length x be affected by change in temperature, such that its refractive index is given by $n(x)$. Resonance condition for the resonator then becomes:

$$n_{eff}(l-x) + \int n(x)dx = m(\mu_n^\lambda + \Delta\mu_n^\lambda) \tag{S36}$$

Removing the constant terms gives

$$\int \left(n(x) - n_{eff}\right)dx = \int \Delta(n(x))dx = m(\Delta\mu_n^\lambda) \tag{S37}$$

Assuming a constant thermo-optic coefficient dn/dT we can write $\Delta n(x) = \rho\Delta T(x)$. Consequently,

$$\int \rho\Delta T(x)dx = m(\Delta\mu_n^\lambda) \tag{S38}$$

This implies shift in onsite potential $\Delta\mu_n^\lambda \propto \int \Delta T(x)dx$.
Next, to estimate this effect of thermal crosstalk in our system and compare it with typically used thermo-optic (TO) heaters we perform a set of thermal simulations using ANSYS Lumerical HEAT (Fig. S6). In Fig. S6(a) we have the schematic depicting a conventional TO heater, where the metallic heating element sits directly on top of the resonator segment separated by a uniform and universal 1.5 μm SiO_2 cladding. In Fig. S6(c) we have the island TO heater design we used for our device. For both these cases we have the CCA made up of only 3 sites with the heater placed on the middle site (n^{th}). We then record the temperature profile in the shorter straight segment of the racetrack resonators (highlighted in yellow) for the nth and $(n+1)^{th}$ sites as we vary the voltage applied V_n across the heater from 0 V to 0.78 V.

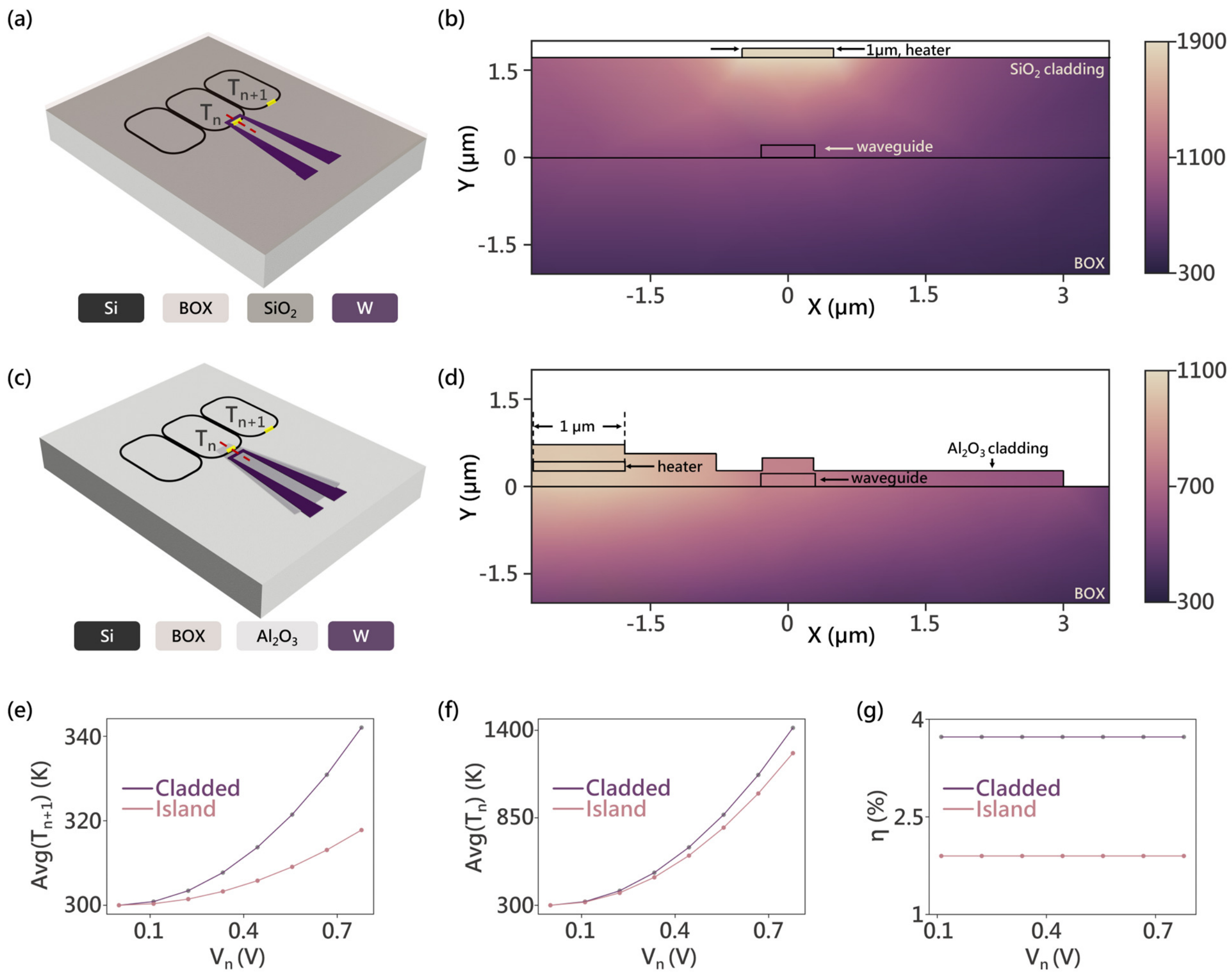

Fig. S6. Thermal simulation results. (a) Schematic depicting the device with conventional TO heater sitting directly over the resonator separated by 1.5 μm thick oxide cladding (label: Cladded). (b) Temperature profile (in K) across the cladded waveguide (cross section denoted by red dashed line in the schematic) when $V_n = 0.56\ V$. (c) Schematic depicting a device equipped with the island TO heater used in our paper (label: Island). (d) Temperature profile (in K) across the island waveguide (cross section denoted by red dashed line in the schematic) when $V_n = 0.56\ V$. (e) Plot comparing average temperature across the straight segment (in yellow) of the $(n+1)^{th}$ resonator. (f) Plot comparing average temperature across the straight segment (in yellow) of the nth resonator. (g) Plot comparing η factor in % for the two device designs. (purple: cladded, pink: island).

Then in Fig. S6(b), (d) we plot temperature profile (in K) across the waveguide cross section (denoted by the red dashed line in the corresponding schematic) for both the designs when 0.56 V is applied across the heater. In Fig. S6(e) we plot the average temperature across the segments given by

$$Avg(T) = \frac{\int T(x)dx}{\int dx} \qquad \text{(S39)}$$

for neighboring $(n+1)^{th}$ site. Similarly, in Fig. S6(f) we plot the average temperature across the segment for n^{th} site. It is clear from these plots that even though both heater designs cause a similar increase in the onsite temperature (n^{th}), the average temperature in the neighboring sites diverges rapidly between the designs with the temperature difference already greater than 24K at $0.78V$. To study the difference in effects of this thermal crosstalk we then define a dimensionless parameter η given by

$$\eta = \frac{\Delta\mu_{n+1}^{\lambda}}{\Delta\mu_{n}^{\lambda}} \tag{S40}$$

Using Eq. S38 we can write the above as

$$\eta = \frac{\Delta\mu_{n+1}^{\lambda}}{\Delta\mu_{n}^{\lambda}} = \frac{\int \Delta T_{n+1}(x)dx}{\int \Delta T_{n}(x)dx} = \frac{\int (T_{n+1}(x) - 300)dx}{\int (T_{n}(x) - 300)dx} \tag{S41}$$

In Fig. S6(g) we plot the η (as %) for both the designs. We can see from the plot that $\eta_{cladded} \sim 0.037$ and $\eta_{island} \sim 0.018$. This implies that the islands TO heaters outperform the typical TO heaters in reducing the effects of thermal crosstalk in the device by $\sim 50\%$.
In general, we do not expect the η values to vary with the voltage V_n (see Section S4) as also verified by the plots.

Section S4: Extracting the thermal crosstalk:

Let voltage V_n be applied across heater h_n at the nth site of the CCA. When only one heater h_n is turned on at time, using Ohmic heating law we can approximate the change in temperature at n^{th} site of the CCA as $Avg(\Delta T_n) \propto V_n^2/R_n$ where R_n is the resistance of heater h_n.
For a constant thermo-optic coefficient dn/dT, from Eq. S38 we know that $\Delta\mu_n^{\lambda} \propto Avg(\Delta T_n)$. Combining the above two relations using a proportionality constant k_1 allows us to write:

$$\Delta\mu_n^{\lambda} = k_1 V_n^2/R_n = \alpha_n V_n^2 \tag{S42}$$

where $\alpha_n = k_1/R_n$.
Since only h_n is turned on, it also follows that for another site m in the array, $\Delta\mu_m^{\lambda} \propto Avg(\Delta T_n)$. Introducing another set of proportionality constants β_{nm} allows us to write:

$$\Delta\mu_m^{\lambda} = \beta_{nm}\alpha_n V_n^2 = \beta'_{nm} V_n^2 \tag{S43}$$

Using Eq. S42, S43 we can then estimate the thermal crosstalk by fitting for these coefficients α_n, β'_{nm} by calculating the change in onsite potentials and estimating the eigen-energies of the modified H_{eff} whose initial state H_{eff}^0 was fitted with the procedure outlined in Section S2 and demonstrated in the paper.

$$[\epsilon_n^{\lambda}]_{estimated} = Eig\left(H_{eff}^0 + [\Delta\mu_n]\mathbb{I}\right), \qquad [\Delta\mu_n^{\lambda}] = [\alpha_n, \beta'_{nm}][V_n^2] \tag{S44}$$

We do this fitting for all the eight heaters $[h_n]$ to obtain corresponding α_ns and β'_{nm}s; accuracy results of which are plotted in Fig. S7. The x-axis denotes the voltage V_n applied to heater h_n, the

y-axis plots the eigen-energies as wavelengths. The black lines in the background denote the measured locations of eigen-energies on application of V_n, the size of the circle denotes the error of the fit from the measured location of eigen-energy. The color of the circles denotes the overall fit error for that particular measurement. The extracted values α_ns and β'_{nm}s are then used to plot Fig. 2 in the paper where we use these to plot the change in $\Delta\mu_n^\lambda$s for all V_ns used in the measurements shown below.

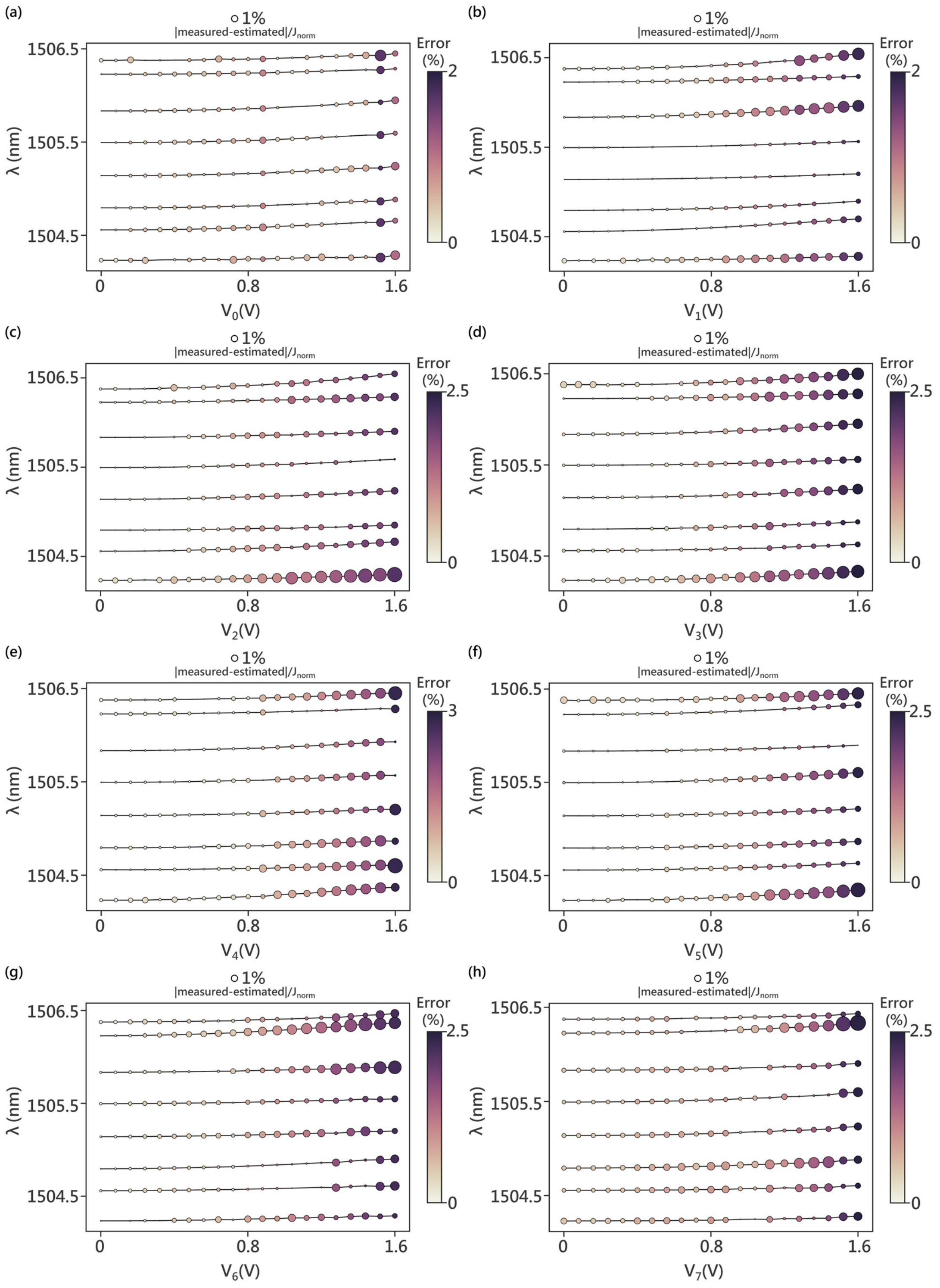

Fig. S7. Thermal crosstalk extraction. Fitting accuracy for sweeps with only one heater turned on at a time where the x-axis denotes the voltage applied V_n, the y-axis denotes the wavelength with the location of the measured eigen-energies is denoted by the dark black lines in background. The radii of the circles denote the deviation of the fitted value from measured values (scale bar on top). The color of the dots denotes the overall prediction error for that generation.

Supplementary references:

1. Little, B. E., Chu, S. T., Haus, H. A., Foresi, J. & Laine, J.-P. Microring resonator channel dropping filters. *Journal of Lightwave Technology* **15**, 998–1005 (1997).
2. Poon, J. K. S., Scheuer, J., Xu, Y. & Yariv, A. Designing coupled-resonator optical waveguide delay lines. *J. Opt. Soc. Am. B, JOSAB* **21**, 1665–1673 (2004).
3. Mittal, S. Topological edge states in silicon photonics. *ProQuest Dissertations and Theses* (University of Maryland, College Park, 2014).
4. Ji, X. *et al.* Compact, spatial-mode-interaction-free, ultralow-loss, nonlinear photonic integrated circuits. *Commun Phys* **5**, 1–9 (2022).
